# Mechanistic Comparison between Gastric Bypass vs. Duodenal Switch with Sleeve Gastrectomy in Rat Models

**DOI:** 10.1371/journal.pone.0072896

**Published:** 2013-09-09

**Authors:** Yosuke Kodama, Helene Johannessen, Marianne W. Furnes, Chun-Mei Zhao, Gjermund Johnsen, Ronald Mårvik, Bård Kulseng, Duan Chen

**Affiliations:** 1 Department of Cancer Research and Molecular Medicine, Norwegian University of Science and Technology, Trondheim, Norway; 2 Department of Surgery, Saint Olav's University Hospital, Trondheim, Norway; University of California, Los Angeles, United States of America

## Abstract

**Background:**

Both gastric bypass (GB) and duodenal switch with sleeve gastrectomy (DS) have been widely used as bariatric surgeries, and DS appears to be superior to GB. The aim of this study was to better understand the mechanisms leading to body weight loss by comparing these two procedures in experimental models of rats.

**Methods:**

Animals were subjected to GB, DS or laparotomy (controls), and monitored by an open-circuit indirect calorimeter composed of comprehensive laboratory animal monitoring system and adiabatic bomb calorimeter.

**Results:**

Body weight loss was greater after DS than GB. Food intake was reduced after DS but not GB. Energy expenditure was increased after either GB or DS. Fecal energy content was increased after DS but not GB.

**Conclusion:**

GB induced body weight loss by increasing energy expenditure, whereas DS induced greater body weight loss by reducing food intake, increasing energy expenditure and causing malabsorption in rat models.

## Introduction

Various bariatric surgical procedures, such as gastric banding, gastric bypass (GB) and duodenal switch with sleeve gastrectomy (DS), have been developed in order to reduce food intake and/or lead to malabsorption [1]. GB was invented by Dr. Edward Mason as a bariatric surgery in 1965, and later it was converted to Roux-en-Y procedure which was created by Dr. Cesar Roux already in 1897. Recently, a laparoscopic mini-GB procedure has been shown to be regarded as a simpler and safer alternative to laparoscopic Roux-en-Y with similar efficacy at 5 or 10 year experience [2]. However, the different procedures have shown different efficacy in individual patients, and the underlying mechanisms are not yet clear. Therefore, it is a challenge to select the most effective bariatric procedure for individual patients.

**Figure 1:**
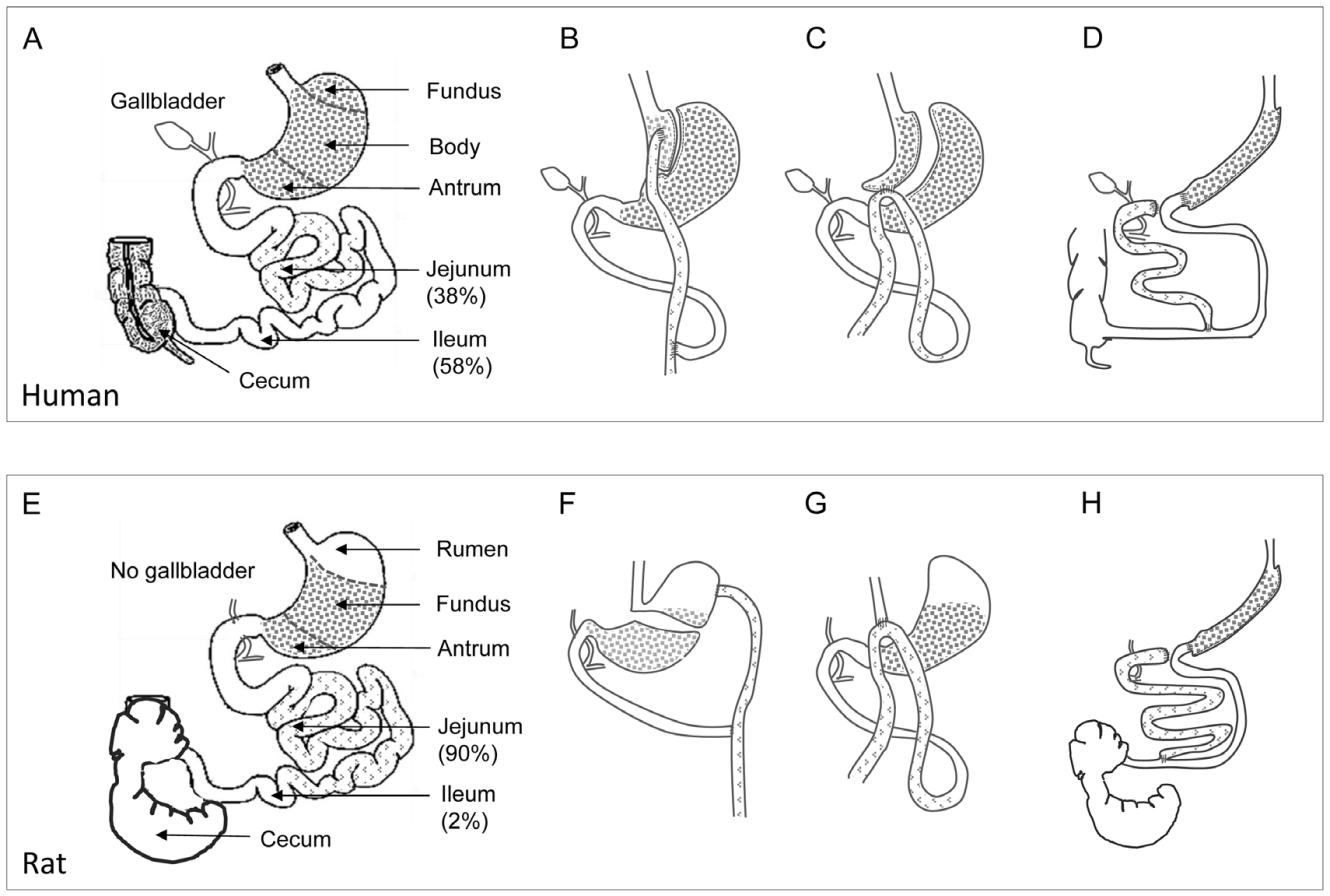
Schematic drawing of anatomy. The gastrointestinal tract of human (**A–D**) and rat (**E–H**) before (**A, E**) and after Roux-en-Y gastric bypass (GB) (**B, F**), mini-GB (**C, G**), and duodenal switch (**D, H**)**.** Glandular stomach is indicated by grid gray and jejunum by light grid gray. The rumen of rat stomach is non-glandular (white area). Note: In **A, E**, percentages mean % of small intestine, e.g. in **E**, jejunum is 90% of total small intestine in rats based on [11]; in **F**, rat Roux-en-Y GB [3], and in **G**, Mini-GB used in the present study.

Various rat models of bariatric surgery have been developed in order to understand the underlying physiological mechanisms of different surgical procedures. There have been many studies in the literature reporting the surgical procedures (such as Roux-en-Y) in rats that are made as same as they are used in humans [3], [4]. However, there is a significant difference in the anatomy and physiology of the gastrointestinal tract between rats and humans, which should be kept in mind when creating the surgical models in rats and translating findings from animals and humans. For instance, the rat stomach consists of antrum, fundus (also called corpus) and rumen (forestomach), while the human stomach is divided into antrum, body and fundus (Fig 1A, E). The rat jejunum represents almost 90% of the small intestine, the human jejunum about 40% [5]. Unlike humans, rats are nonemetic (not vomiting) and has no gallbladder. The Roux-en-Y reconstruction was initially created to prevent post-gastrectomy bile vomiting in patients [6]. Apparently, it is not necessary to create the Roux-en-Y reconstruction in rats that are subjected to GB [7]–[9]. The duodenal switch procedure was originally created as a surgical solution for primary bile reflux gastritis and/or to decrease postoperative symptoms after distal gastrectomy and gastroduodenostomy [10]. In patients, the operation usually consists of a 75% longitudinal gastrectomy (the so-called sleeve gastrectomy), creation of an alimentary limb approximately 50% of total small bowel length (i.e. bypassing jejunum), a common channel length of 100 cm, and cholecystectomy. In the present study using rats, GB was performed without the Roux-en-Y reconstruction and the postsurgical anatomy was similar to mini-GB on humans, and DS was performed according to the rat anatomy (Fig 1A–H) [11].

GB is the most common procedure because of relatively high efficacy and safety, whereas DS seems to be even more effective, particularly in super-obese patients [12]. Both GB and DS are believed to cause restriction in food intake and malabsorption by decreasing stomach size and bypassing part of the small intestine. In patients, DS is superior to GB in body weight loss as well as in improvement of comorbidities such as diabetes, hypertension and dyslipidemia [12]–[18]. Mechanisms underlying the postoperative weight loss and possible regain remain unclear. Whether this is due to biological or behavioral factors is one of the major debates [19]. The aim of the present study was to compare the postoperative effects of GB *vs.*DS on eating behavior and energy expenditure in rat models.

## Materials and Methods

### Animals and Experimental Design

**Figure 2:**
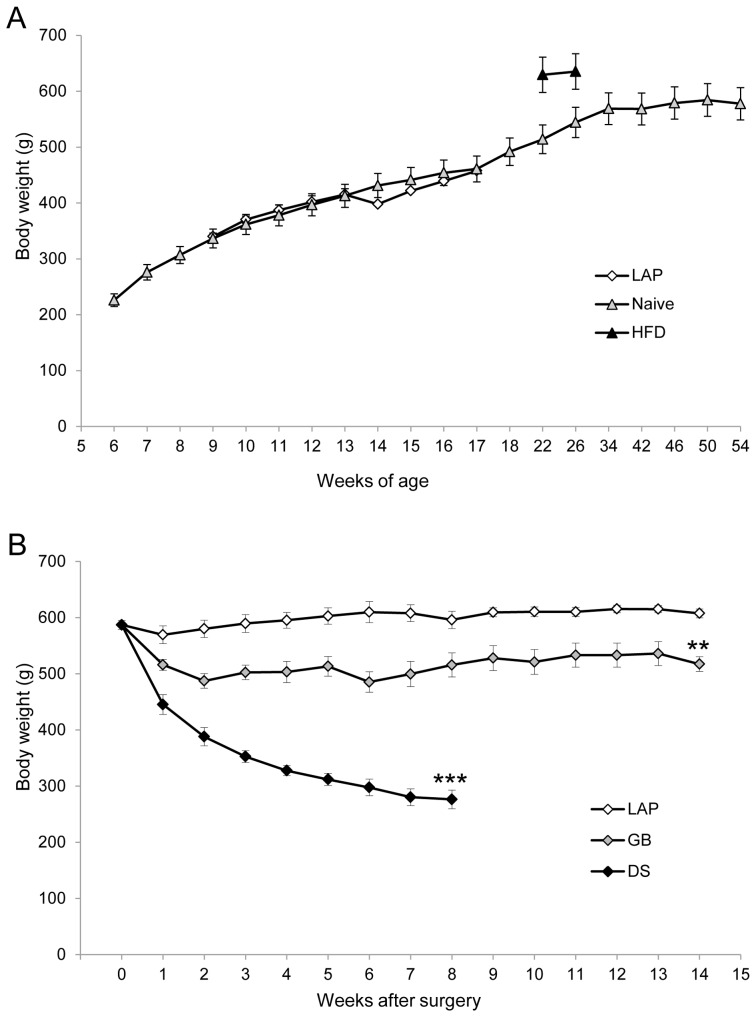
Body weight. Naïve rats (data from Taconic), rats that underwent laparotomy (LAP) at 13 weeks (LAP) and rats that have had high-fat since 5 weeks of age (data from [31]) (**A**)**.** Rats after gastric bypass (GB), duodenal switch (DS) and laparotomy (LAP) (**B**). Data are expressed as means ± SEM. **: *p*<0.01, ***: *p*<0.001 between LAP *vs.* GB or DS.

Adult rats (male, Sprague-Dawley, 6–12 months of age) were purchased from Taconic M&B, Skensved, Denmark and housed in ventilated cages in a specific pathogen free environment with room temperature of 22°C, 40–60% relative humidity and 12 hr day/night cycle with 1 hr dusk/dawn. The rats had free access to tap water and standard rat pellet food (RM1 801002, Scanbur BK AS, Sweden). In our previous studies, we have reported that the male rats gained body weight mainly as a result of continuous expansion of the fat compartment after puberty (8 weeks of age with 200 g body weight), and that the male rats that were fed a high-fat diet starting at 5 weeks of age gained body weight up to ∼650 g at 40 weeks of age as a result of increased fat mass [7], [8]. In the present study, normal adult male rats (∼600 g body weight) were chosen after considerations of the small difference in body weight (∼650 g *vs.* ∼600 g induced by high-fat diet) and the experimental efforts in terms of time-consuming and financial expense (Fig 2A). Furthermore, the body weight development of naïve rats reaches a plateau (580±20 g) at 40 weeks of age, and laparotomy performed at 13 weeks of age did not affect the development of body weight (Fig 2A).

Thirty-four rats, at 587.0±8.1 g body weight, were randomly divided into experimental (GB and DS) as well as control groups (laparotomy, LAP): GB (14 rats), DS (7 rats), and LAP (13 rats). The body weight was not different between the groups before surgery (*p* = 0.276). Because of markedly loss of body weight after DS, the group of DS rats, together with age-matched group of laparotomized rats (LAP_DS_, 7 rats), were followed up only for 8 weeks, while GB rats and the rest of laparotomized rats (LAP_GB_, 6 rats) were followed up for 14 weeks. In consideration of the “3Rs” for the human use of animals (i.e., reducing the number of animals while achieving the scientific purposes of the experiment), rats that had been used for studies of the effects of individual surgical procedures were re-used [8], [20]. The study was approved by the Norwegian National Animal Research Authority (Forsøksdyrutvalget, FDU).

### Surgery

All operations were performed under general anesthesia with isofluran (4% for induction and 2% for maintenance) (Baxter Medical AB, Kista, Sweden). Buprenorphine (0.05 mg/kg) (Schering-Plough Europe, Brussels, Belgium) was administrated as an analgesic agent subcutaneously immediately during surgery. LAP was performed through a middle-line incision with gentle manipulation of viscera. A rat model of Roux-en-Y GB procedure has been described by Stylopoulos and his colleagues [3]. Gastric pouch in that rat model was created at the site of rumen which does not exist in humans (Fig 1F). In the present study, GB was performed by anastomosing the distal esophagus to the proximal jejunum about 2–3 cm distal to the Treitz ligament in an end-to-side manner (Fig 1G) as described previously [7], [8]. DS was achieved in two stages. The two-stage procedure has been recommended in patients because the single-stage procedure increases the risk of postoperative complications and staged DS may avoid biliopancreatic diversion in some patients [21]. In the present study, sleeve gastrectomy was performed by resecting approximately 90% of the rumen and 70% of the glandular stomach along the greater curvature. Three months later, duodenal switch was achieved by creating biliopancreatic limb, alimentary limb (bypassing jejunum) and common channel length of 5 cm (Fig 1H). The duration of surgical time was 30–60 min for GB or DS. In all surgeries performed in the present study, proper aseptic surgical techniques were applied, and therefore, neither prophylactic nor postoperative antibiotics were used. This was done according to the guidelines and recommendations by the Federation of European Laboratory Animal Science Associations (FELASA 2008) and the guide for the care and use of laboratory animals by the Committee of USA National Research Council (2010). After recovering from anesthesia, the animals were placed 2–4 per cage throughout the study period.

### Measurements of Eating Behavior and Energy Expenditure Parameters

These were monitored by the Comprehensive Laboratory Animal Monitoring System (CLAMS, Columbus Instruments International, Columbus, OH, USA) 2–3 weeks after GB, DS or LAP and 14 weeks after GB, 8 weeks after DS or 8–14 weeks after LAP. The CLAMS is composed of a 4-chamber indirect calorimeter designed for the continuous monitoring of individual rats from each chamber. The eating data was generated by monitoring all feed balances every 0.5 s. In CLAMS program used in the present study, the end of an eating event (meal) was when the balance was stable for more than 10 s and a minimum of 0.05 g was eaten. The eating parameters during daytime and nighttime (12 hr each time) for each rat included: accumulated food intake (g or kcal), number of meals, meal size (g/meal), meal duration (min/meal), intermeal interval (min), rate of eating (g/min), and satiety ratio (min/g). The intermeal interval was defined as the interval in minutes between two meals. The rate of eating was calculated by dividing meal size by meal duration. The satiety ratio, an index of non-eating time produced by each gram of food consumed, was calculated as intermeal interval divided by meal size [22]. The volume of O_2_ consumption (VO_2_ mL/kg/hr) and the volume of CO_2_ production (VCO_2_ mL/kg/hr) were measured by an air sample withdrawn every 5 min from each chamber through the gas dryer. The energy expenditure (kcal/hr) was calculated according to equation: (3.815+1.232 RER) × VO_2_, where the respiratory exchange ratio (RER) was obtained by VCO_2_ divided by VO_2_. In order for rats to acclimate to CLAMS, they were placed in these metabolic chambers for 24 hr one week before the first CLAMS monitoring. For the measurement of eating and metabolic parameters, the rats were placed in the CLAMS for 48 hr. In order to minimalize possible effect of stress, only data from the last 24 hr in CLAMS were used for the analysis. An analysis of eating pattern in control rats over a time period from day 1 and 21 showed no significant differences in any parameters, indicating that the animals had acclimated to CLAMS (Table S1). The rats have had free access to standard rat powder food (RM1 811004, Scanbur BK AS, Sweden) and tap water while they were in CLAMS. The total metabolizable energy was 2.57 kcal/g for both the pellet food (RM1 811002) and the powder food (RM1 811004).

### Determination of Fecal Energy Density

Feces were collected while the rats were placed in CLAMS chambers and dried for 72 hr at 60°C. The energy density was determined by means of an adiabatic bomb calorimeter (IKA-Calorimeter C 5000, IKA-Werke GmbH & Co. KG, Staufen, Germany).

### Determination of Plasma Levels of Cytokines

Blood was drawn from the abdominal aorta under the anesthesia just before the animals were killed, and plasma was stored at −80°C until determination of levels of cytokines. The multiplex cytokine assay was used (Cat no:171-K1002M, Bio-plex Pro Rat Cytokine Th1/Th2 12-plex Panel; Bio-Rad Laboratories, Hercules, CA, USA). It contained the following analytics: IL-1α, IL-1β, IL-2, IL-4, IL-5, IL-6, IL-10, IL-13, granulocyte-macrophage colony stimulating factor (GM-CSF), interferon gamma (IFNγ), and tumor necrosis factor alpha (TNFα).

### Statistical Analysis

The values were expressed as means ± SEM. Two-tailed independent-samples *t*-test or Mann Whitney U test was performed for two-group comparisons. ANOVA followed by Bonferroni test was performed for multiple comparisons. Homogeneity of regression assumption test and ANCOVA were performed for analysis of energy expenditure. SPSS version 19.0 (SPSS Inc. Chicago, IL, USA) was used. A *p*-value of <0.05 was considered statistically significant.

## Results

### Mortality

No one died after LAP alone, 6 after GB, and 2 after DS due to surgical complications, trauma and learning curve factors.

### Body Weight

LAP alone did not reduce body weight during the study period (maximum 14 weeks). GB caused approximately 20% weight loss throughout the study period (14 weeks). DS induced approximately 50% weight loss within 8 weeks (Fig 2B).

### Food Intake and Eating Behavior

**Figure 3:**
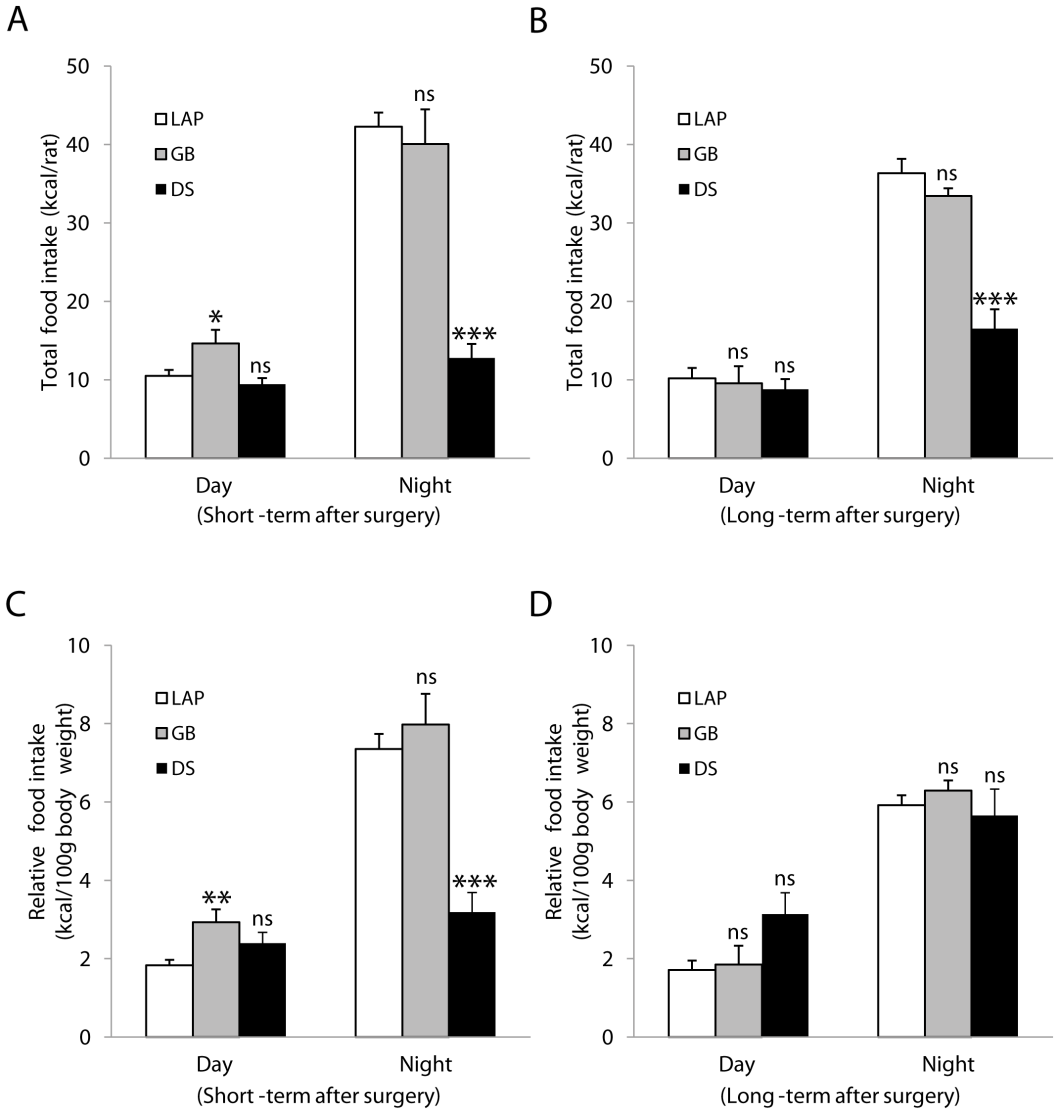
Food intake. Total food intake (kcal/rat) (**A,B**) and relative food intake (kcal/100 g body weight) (**C,D**) during day- and night-time. Short-term after surgery: 3 weeks after gastric bypass (GB), 2 weeks after duodenal switch (DS) or 2–3 weeks after lapatoromy (LAP). Long-term after surgery: 14 weeks after GB, 8 weeks after DS or 8–14 weeks after LAP. Data are expressed as means ± SEM. *: *p*<0.05, **: *p*<0.01, ns: not significant between LAP (n = 13) *vs.* GB (n = 8) or DS (n = 5).

In comparison with LAP, GB increased daytime (but not nighttime) food intake (expressed as either kcal/rat or kcal/100 g body weight) at 3 weeks, and had no effects afterwards (14 weeks postoperatively). In contrast, DS reduced nighttime (but not daytime) food intake (kcal/rat at both 2 and 8 weeks or kcal/100 g body weight at 2 weeks). The food intake (kcal/100 g) at 8 weeks was not reduced because of markedly loss of the body weight after DS (Fig 3).

**Figure 4:**
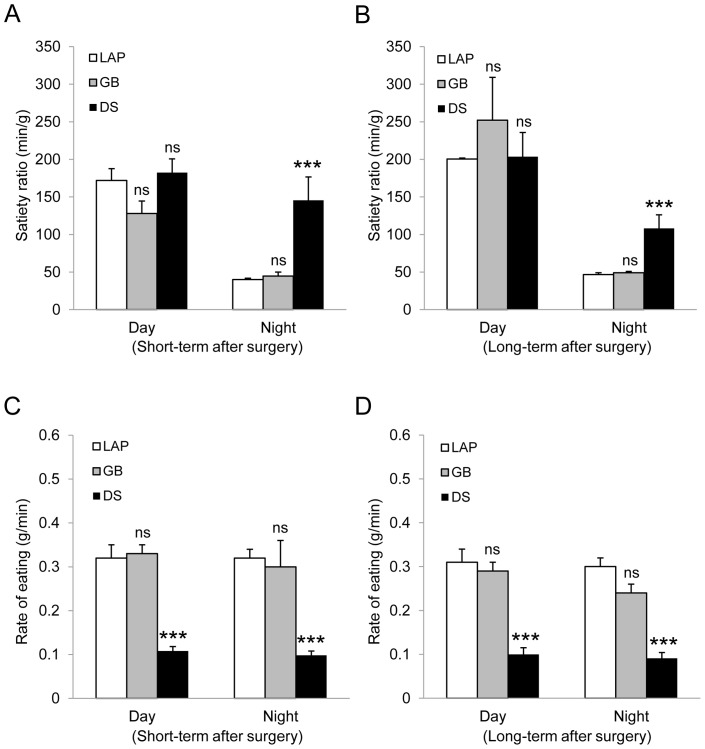
Eating behavior. Satiety ratio (min/g) (**A,B**) and rate of eating (g/min) (**C,D**) during day- and night-time**.** Short-term after surgery: 3 weeks after gastric bypass (GB), 2 weeks after duodenal switch (DS) or 2–3 weeks after lapatoromy (LAP). Long-term after surgery: 14 weeks after GB, 8 weeks after DS or 8–14 weeks after LAP. Data are expressed as means ± SEM. ***: *p*<0.001, ns: not significant between LAP (n = 13) *vs.* GB (n = 8) or DS (n = 5).

GB was without effects neither on satiety ratio (min/g) nor rate of eating (g/min), whereas DS increased satiety ratio during nighttime, and decreased rate of eating during both daytime and nighttime at 2 weeks and 8 weeks postoperatively (Fig 4) (Tables 1,2).

**Table 1:** Eating behavior.

	Parameter	LAP	GB	DS
**Day**	Food Intake (g)	4.08±0.3	5.69±0.68*	3.67±0.31^†^
	Food Intake (g/100g body weight)	0.7±0.06	1.14±0.13**	0.93±0.11
	Food intake (kcal)	10.5±0.76	14.62±1.74*	9.42±0.80^†^
	Food intake (kcal/100g body weight)	1.83±0.14	2.93±0.33**	2.39±0.28
	Number of meals	12.15±0.97	18.29±2.48*	16.6±0.92
	Meal size (g/meal)	0.35±0.03	0.32±0.02	0.22±0.021*
	Meal size (kcal/meal)	0.91±0.08	0.82±0.05	0.57±0.05*
	Meal duration (min)	13.5±1.39	17.96±2.82	34.47±3.29***,^†††^
	Meal duration (min/meal)	1.14±0.1	0.98±0.08	2.13±0.29***,^†††^
	Intermeal interval (min)	57.5±4.42	40.37±5.38*	39.38±2.04
	Satiety ratio (min/g)	171.89±15.81	127.93±16.63	182.34±18.24
	Rate of eating (g/min)	0.32±0.03	0.33±0.02	0.11±0.01***,^†††^
**Night**	Food Intake (g)	16.45±0.69	15.58±1.72	4.96±0.70***,^†††^
	Food Intake (g/100g body weight)	2.86±0.15	3.11±0.30	1.24±0.19***,^†††^
	Food intake (kcal)	42.28±1.77	40.05±4.41	12.76±1.81***,^†††^
	Food intake (kcal/100g body weight)	7.35±0.39	7.98±0.78	3.19±0.50***,^†††^
	Number of meals	34.38±3.92	31.71±3.01	24.40±3.85
	Meal size (g/meal)	0.57±0.09	0.51±0.07	0.21±0.03*
	Meal size (kcal/meal)	1.47±0.22	1.30±0.17	0.55±0.08*
	Meal duration (min)	54.21±4.54	56.25±5.13	53.21±9.39
	Meal duration (min/meal)	1.88±0.36	1.82±0.18	2.20±0.28
	Intermeal interval (min)	22.6±3.08	21.36±1.97	29.43±5.57
	Satiety ratio (min/g)	40±1.86	44.64±5.41	145.51±31.17***,^†††^
	Rate of eating (g/min)	0.32±0.02	0.30±0.06	0.10±0.01***,^††^

Parameters during day- and night-time at 3 weeks after gastric bypass (GB), 2 weeks after duodenal switch (DS) and 2–3 weeks after laparotomy (LAP). Data are expressed as means ± SEM. *: *p*<0.05, **: *p*<0.01, ***: *p*<0.001 between LAP *vs.* GB or DS. ^†^: *p*<0.05, ^††^: *p*<0.01, ^†††^: *p*<0.001 between GB *vs.* DS.

**Table 2:** Eating behavior.

	Parameter	LAP	GB	DS
**Day**	Food Intake (g)	3.97±0.51	3.71±0.85	3.42±0.50
	Food Intake (g/100g body weight)	0.66±0.09	0.72±0.19	1.22±0.21
	Food intake (kcal)	10.19±1.32	9.54±2.18	8.78±1.29
	Food intake (kcal/100g body weight)	1.71±0.24	1.85±0.48	3.14±0.54
	Number of meals	10.92±1.29	11.25±1.81	18.60±4.50
	Meal size (g/meal)	0.4±0.06	0.34±0.06	0.23±0.05
	Meal size (kcal/meal)	1.03±0.17	0.86±0.15	0.59±0.12
	Meal duration (min)	13.54±1.74	13.89±3.86	37.17±6.92***,^†††^
	Meal duration (min/meal)	1.37±0.22	1.23±0.22	2.34±0.45
	Intermeal interval (min)	68.39±7.92	73.68±16.91	50.16±17.90
	Satiety ratio (min/g)	200.34±27.9	252.16±57.01	203.50±32.22
	Rate of eating (g/min)	0.31±0.03	0.29±0.02	0.10±0.01***
**Night**	Food Intake (g)	14.15±0.71	13.00±0.38	6.43±0.96***,^†††^
	Food Intake (g/100g body weight)	2.3±0.1	2.45±0.10	2.20±0.26
	Food intake (kcal)	36.35±1.82	33.42±0.98	16.52±2.46***,^†††^
	Food intake (kcal/100g body weight)	5.92±0.25	6.29±0.26	5.65±0.68
	Number of meals	26.92±2.76	26.50±3.69	34.20±7.13
	Meal size (g/meal)	0.59±0.06	0.58±0.09	0.24±0.07*,^†^
	Meal size (kcal/meal)	1.52±0.15	1.48±0.23	0.61±0.19*,^†^
	Meal duration (min)	50.66±4.57	56.36±2.96	70.58±5.38*
	Meal duration (min/meal)	2.01±0.18	2.53±0.47	2.39±0.44
	Intermeal interval (min)	27.78±3.84	27.91±4.11	21.49±4.03
	Satiety ratio (min/g)	46.78±2.4	49.20±1.56	108.18±17.98***,^†††^
	Rate of eating (g/min)	0.3±0.02	0.24±0.02	0.09±0.01***,^††^

Parameters during day- and night-time at 14 weeks after gastric bypass (GB), 8 weeks after duodenal switch (DS) and 8–14 weeks after laparotomy (LAP). Data are expressed as mean ± SEM. *: *p*<0.05, **: *p*<0.01, ***: *p*<0.001 between LAP *vs.* GB or DS. ^†^: *p*<0.05, ^††^: *p*<0.01, ^†††^: *p*<0.001 between GB *vs.* DS.

### Energy Expenditure

**Figure 5:**
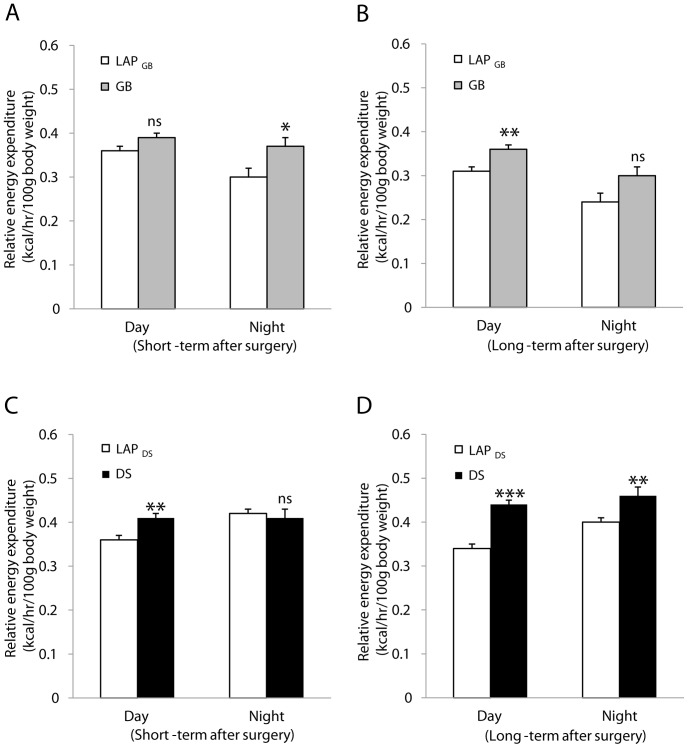
Energy expenditure during day- and night-time. Short-term after surgery: 3 weeks after gastric bypass (GB), 2 weeks after duodenal switch (DS) or 2–3 weeks after laparotomy (LAP). Long-term after surgery: 14 weeks after GB, 8 weeks after DS or 8–14 weeks after LAP. Data are expressed as means ± SEM. *: *p*<0.05, **: *p*<0.01, ***: *p*<0.001, ns: not significant between LAP_GB_ (n = 7) *vs.* GB (n = 8) or LAP_DS_ (n = 6) *vs.* DS (n = 5).

Age-matched control rats that underwent LAP only were included for comparisons because metabolic parameters are age-dependent [23], [24]. GB increased nighttime energy expenditure (kcal/hr/100 g body weight) at 3 weeks and daytime energy expenditure at 14 weeks postoperatively (Fig 5A, C) (Tables 3, 4). RER was unchanged after GB. DS increased daytime energy expenditure both at 2 and 8 weeks as well as nighttime energy expenditure at 8 weeks postoperatively (Fig 5B, D) (Tables 3, 4). RER tended to be reduced during nighttime at 2 weeks after DS (*p* = 0.051) (Table 3).

**Table 3:** Metabolism.

		3 weeks after surgery		2 weeks after surgery	
	Parameter	LAP_GB_	GB	LAP_DS_	DS
**Day**	Energy expenditure (kcal/hr)	2.08±0.03	1.93±0.06	2.08±0.07	1.66±0.12**
	Energy expenditure (kcal/hr/100g body weight)	0.36±0.01	0.39±0.01	0.36±0.01	0.41±0.01**
	Energy expenditure (kcal/hr/cm^2^ body surface)	0.003±0.0001	0.004±0.0001	0.003±0.0001	0.004±0.0002
	RER	0.94±0.01	0.93±0.02	1.02±0.03	0.96±0.03
	VO_2_	726.68±15.56	779.91±23.26	705.18±18.00	823.91±31.49**
	VCO_2_	682.50±12.08	727.85±23.54	717.08±26.45	791.69±26.59
**Night**	Energy expenditure (kcal/hr)	1.75±0.14	1.85±0.10	2.45±0.08	1.66±0.10***
	Energy expenditure (kcal/hr/100g body weight)	0.30±0.02	0.37±0.02*	0.42±0.01	0.41±0.02
	Energy expenditure (kcal/hr/cm^2^ body surface)	0.003±0.0002	0.003±0.0002	0.004±0.0001	0.004±0.0002*
	RER	1.00±0.01	1.01±0.03	1.07±0.03	0.97±0.04
	VO_2_	600.02±36.70	737.88±43.20*	820.65±20.88	823.94±35.51
	VCO_2_	594.19±37.54	735.07±35.39*	877.73±35.71	803.06±49.36

Parameters during day- and night-time at 3 weeks after gastric bypass (GB) and the age-matched laparotomy-operated group (LAP_GB_), and at 2 weeks after duodenal switch (DS) and the age-matched laparotomy-operated group (LAP_DS_). Data are expressed as means ± SEM. *: *p*<0.05, **: *p*<0.01, ***: *p*<0.001 between LAP_GB_ *vs.* GB or LAP_DS_ *vs.* DS.

**Table 4:** Metabolism.

		14 weeks after surgery		8 weeks after surgery	
	Parameter	LAP_GB_	GB	LAP_DS_	DS
**Day**	Energy expenditure (kcal/hr)	1.92±0.08	1.90±0.05	2.10±0.06	1.27±0.07***
	Energy expenditure (kcal/hr/100g body weight)	0.31±0.01	0.36±0.01**	0.34±0.01	0.44±0.01***
	Energy expenditure (kcal/hr/cm^2^ body surface)	0.003±0.0001	0.003±0.0000*	0.003±0.0000	0.003±0.0001
	RER	0.98±0.03	0.91±0.05	0.99±0.03	0.98±0.01
	VO_2_	622.25±27.61	720.95±14.41**	681.99±13.62	873.32±25.35***
	VCO_2_	605.31±17.73	652.48±25.93	675.60±23.89	853.15±25.17***
**Night**	Energy expenditure (kcal/hr)	1.48±0.11	1.60±0.10	2.45±0.12	1.34±0.01***
	Energy expenditure (kcal/hr/100g body weight)	0.24±0.02	0.30±0.02	0.40±0.01	0.46±0.02***
	Energy expenditure (kcal/hr/cm^2^ body surface)	0.002±0.0002	0.003±0.0002	0.004±0.0001	0.004±0.0001*
	RER	0.97±0.06	1.04±0.05	1.06±0.03	0.99±0.03
	VO_2_	475.14±36.56	598.36±45.29	778.37±16.51	919.50±28.99**
	VCO_2_	487.63±35.15	609.64±55.95	825.43±27.97	906.74±39.86

Parameters during day- and nighttime at 14 weeks after gastric bypass (GB) and the age-matched laparotomy-operated group (LAP_GB_), and at 8 weeks after duodenal switch (DS) and the age-matched laparotomy-operated group (LAP_DS_). Data are expressed as means ± SEM. *: *p*<0.05, **: *p*<0.01, ***: *p*<0.001 between LAP_GB_ *vs.* GB or LAP_DS_ *vs.* DS.

Analysis of the homogeneity of regression slopes indicated that there was positive correlation between the body weight and energy expenditure (kcal/hr) particularly in LAP_DS_ rats and similar regression slopes between LAP and GB or DS (*p*>0.05) (Fig S1). ANCOVA showed that there was no significant difference in adjusted energy expenditure between LAP and GB or DS (*p*>0.05) (Fig S2).

### Fecal Energy Density

**Figure 6:**
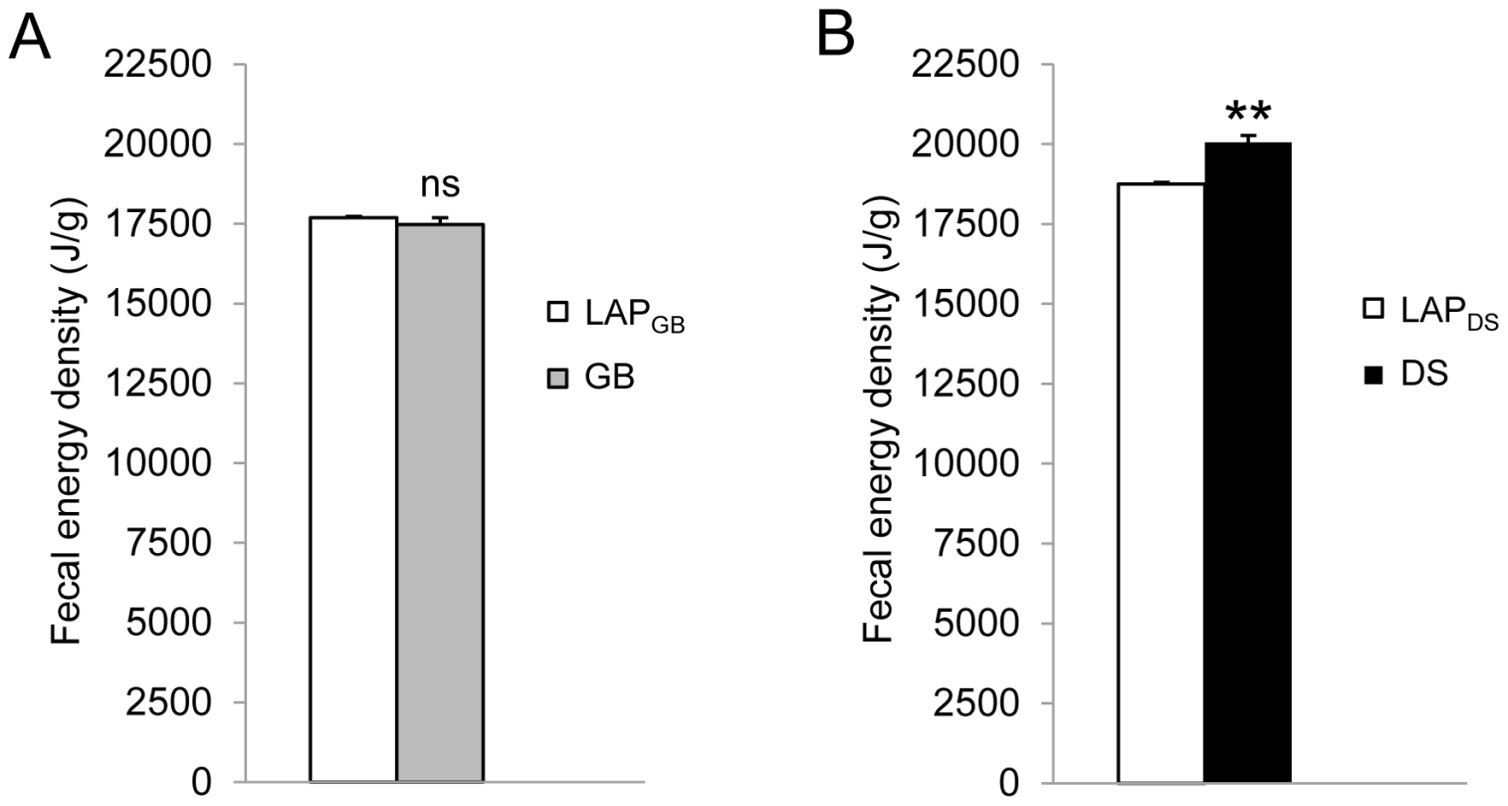
Fecal energy density. Three weeks after gastric bypass (GB) or laparotomy (LAP_GB_) (**A**) and eight weeks after duodenal switch (DS) or laparotomy (LAP_DS_) (**B**). Data are expressed as mean ± SEM. **: *p*<0.01, ns: not significant between LAP_GB_ (n = 7) *vs.* GB (n = 8) or LAP_DS_ (n = 6) *vs.* DS (n = 5).

There was no change in the fecal energy density after GB. DS had severe diarrhea within 2 weeks postoperatively, so that it was difficult to collect the fecal samples. At 2 months, the solid feces were collected and the energy density was increased (Fig 6).

### Plasma Levels of Cytokines

There was no difference between LAP *vs.* GB or DS in the plasma levels of the 11 cytokines measured (Table S2).

## Discussion

The present study shows that the rat models provide results that are in accordance with results from clinical series in patients , i.e. greater weight loss by DS than GB [17], [18], [25]. Furthermore, the results of the present study show different postsurgical effects of GB *vs.* DS in terms of food intake, eating rate, energy expenditure and absorption.

It is a common dogma that to reduce size of stomach by surgery would lead to early satiety and consequently reduce food intake. Regardless of difference in surgical procedure, either GB or DS reduces stomach size and bypasses part of the small intestine (duodenum and most of the jejunum). Previously, we have found that the food intake was independent on the size of stomach by comparing gastrectomy and GB in rat models [9]. In clinical studies, the size of pouch after GB was found not to correlate with weight loss outcome in patients [25], [26]. In our previous and the present studies, GB did reduce body weight but not food intake in rats [7]–[9]. Behavior of rats is mostly driven on instincts, while behavior of humans is much more complicated. In fact, there is still an open question: “Does GB reduce food intake in humans?”. A recent review shows that large and persistent alterations in macronutrient intake after GB have not generally been reported, and when the changes do occur, they are either transient or relatively modest. The authors argue for more direct measures of food intake in human studies that are similar to those used in animal studies [27]. Food intake in patients is also affected by following the “postoperative instruction” to achieve the best possible conditions for weight reduction and to minimize side-effects like gastro-esophageal reflux and dumping syndrome which unlikely occur in rats. Recently, a human study of eating behavior and meal pattern following GB was still performed by manually weighing differences to determine food and water intake and by the Three-Factor Eating Questionnaire to evaluate eating behavior [28]. However, in that human study, the food intake was not reported, but *ad libitum* meal size was reduced while number of meals per day was increased, and hunger and satiety scores did not change after GB, which are in line with our findings in rats following GB [8]. Methods with more direct measures of food intake (and food-selection and taste-related behavior) for humans are needed in order to facilitate translation between findings from animal models and clinical research [27].

Unlike GB, DS does reduce the food intake. Previously, we have shown that food intake was reduced by duodenal switch alone but not by sleeve gastrectomy alone by comparing sleeve gastrectomy only *vs.* duodenal switch without sleeve gastrectomy in rats [20]. In the present study, DS markedly reduced food intake and increased satiety ratio particularly during nighttime. The rate of eating has also impacts on body weight. It has been reported that there is a correlation between rate of eating and body weight or body mass index (BMI) [29], [30]. Previously, we have shown that high-fat-diet-induced obesity was associated with increased rate of eating, increased size of meals, but not with daily calories intake [31]. In the present study, DS decreased the eating rate during both day- and night-time.

Mechanisms underlying postoperative weight loss and possible regain remain unclear. A major point of controversy is whether this is due to biological or behavioral factors [19], [32]. We and others have shown that GB increased the energy expenditure in rats and mice, which could be one of the mechanisms explaining the physiologic basis of weight loss after this procedure [8], [33], [34]. The increased resting energy expenditure in the animal models after GB is in accordance with some, but not all, reports in humans. The discrepancies in the clinical studies may include the heterogeneity of patient populations and measurements of energy expenditure for a limited time using portable metabolic carts under artificial rather than “free-living” conditions [35]. Nerveless, resting energy expenditure has been suggested to be a therapeutic target for obesity [32], [36]. In the present study, we further showed that the increased energy expenditure took place only during nighttime (relevant to active energy expenditure) shortly after GB (weeks) and switched to daytime (resting energy expenditure) after months, whereas the energy expenditure was increased during daytime shortly after DS and during both day- and night-time months after DS.

The most extensively used method for calculation of energy expenditure is dividing O_2_ consumption by body weight or body surface area [37]. In the present study, the energy expenditure was calculated by taking into account both O_2_ consumption and CO_2_ production, and expressed as kcal/hr/rat, kcal/hr/100 g body weight, and kcal/hr/cm^2^ body surface. Dividing the energy expenditure by body weight does not take into account differences in body composition, and therefore, the fat-free mass or lean body mass (as denominator) has often been used in both human and mouse studies. However, this could be inappropriate because brown fat can be the most metabolically active tissue in the body [37]. ANCOVA has been suggested to be appropriate method for analysis of the mouse energy expenditure, but it cannot be used when the samples sizes are small [37], [38]. In rat studies, ANCOVA has not been used with the exception of a few reports including our previous study of ileal interposition associated with sleeve gastrectomy [5], [39]. The reasons for not widely use of ANCOVA than ANOVA in rat studies might be less statistical power when sample size is small and nonlinear relationship between covariate (s) and dependent variable. Another reason may be that ANCOVA is best used with quasi-experimental data, such as genetically-modified mice [37] or humans [40], [41]. The results of the present study showed that there were highly correlation between the body weight and the energy expenditure (kcal/hr/rat) in control LAP rats, and significant increases in the energy expenditure (kcal/hr and/or kcal/hr/100 g body weight) after GB or DS (by ANOVA). However, ANCOVA showed no significant difference in the energy expenditure (kcal/hr) between LAP and GB or DS. The difference in terms of *p* values by ANOVA *vs.* ANCOVA (i.e. testing the body-weight independent differences) can be interpreted as that GB or DS increases the energy expenditure (possible cause) while reducing the body weight (effect), which is at odds with the positive correlation between the body weight and the energy expenditure in control animals (LAP).

Both GB and DS are designed for restriction and malabsorption by creating the alimentary limb. However, DS, but not GB, caused diarrhea shortly after surgery (2 weeks) and malabsorption (measured at 2 months postoperatively) in rats, which is in line with observations in patients [42].

It should be noticed that in the present study, neither prophylactic nor postoperative antibiotics were used and none of the 11 plasmas cytokines measured was changed after surgery, indicating no or little impact of microflora and inflammation on the eating behavior and the body weight changes. Recently, a mouse study showed that specific alterations in the gut microbiota contributed to the beneficial effect of bariatric surgery on energy balance [43]. Whether GB or DS alters the gut microbiota and consequently leads to the weight loss via same or different pathways might be of interest for future study.

There are several limitations of the present study. 1) The rats used were not obese. Whether or not the postsurgical effects of these two procedures are different between normal and obese rats that are induced by high-fat diet or developed spontaneously (e.g. Zucker, Otsuka Long-Evans Tokushima Fatty, Obese SHR, or Wistar Ottawa Karlsburg W rats), and which animal model of obesity best mimics the obese humans in response to the bariatric surgery could be the subjects for further research. 2) GB procedure used in rats was not exactly the same as it was applied in humans. Fig 1 shows different procedures of GB. A laparoscopic mini-gastric bypass procedure (which is similar with one used in the present study) has been shown to be regarded as a simpler and safer alternative to laparoscopic Roux-en-Y procedure with similar efficacy at 5 or 10 year experience [2], [44]. It may be of interest to compare different GB procedures in the future, if there is any clinical relevancy. 3) Although the size of gastric pouch after GB does not correlate with weight loss outcome in patients [25], [26], it cannot be excluded whether lack of the pouch in GB has impact on food intake, satiety and eating behavior. 4) The differences between rats and humans are not only in terms of the GI anatomy but also the responses to surgery. For instance, sleeve gastrectomy only (without duodenal switch) works in some patients but not in rats [20], [45]. It may be of interest to directly compare the effects of sleeve only *vs.* sleeve with duodenal switch (one or two-staged) in the future.

In general, research in patients is directly clinical relevant. However, studies in animals provide much greater latitude in control and experimental manipulation of the system, and ultimately help to reveal the underlying mechanisms and to adopt the protocols and methods that are tested in animals to humans [46]. Research using animal models is an excellent way of developing and learning bariatric surgical techniques as well as understanding the postsurgical physiology [47]. Taken the data from the previous and the present studies together, the appropriately designed rat models provide significant insights into the mechanisms of bariatric surgery which explain well the clinical observations, e.g. that DS is superior to GB in body weight loss. The results of the present study may suggest further that GB induces body weight loss by increasing energy expenditure, whereas DS induces greater body weight loss by reducing food intake, increasing energy expenditure and causing malabsorption.

## Supporting Information

Figure S1 **Scatterplot of energy expenditure against body weight.** LAP_GB or DS_: laparotomy as control for GB or DS; GS: gastric bypass; DS: Duodenal switch.
(TIF)

Figure S2 **Adjust energy expenditure by ANCOVA.** LAP_GB or DS_: laparotomy as control for GB or DS; GS: gastric bypass; DS: Duodenal switch. Means ± SEM.
(TIF)

Table S1 **CLAMS measurements of normal rats.** Data at day 1 and 21 one week after 24 hours training with CLAMS cage are expressed as means ± SEM. ns: not significant between day 1 vs. day 21.
(DOC)

Table S2 **Plasma levels of cytokines.** Data of rats after gastric bypass (GB) and duodenal switch (DS) compared with the age-matched laparotomy-operated groups (LAP_GB_ or LAP_DS_, respectively) are expressed as means ± SEM. ns: not significant between LAP_GB_ vs. GB or LAP_DS_ vs. DS.
(DOC)
