# Supplementary figures and images for: Mechanistic Comparison between Gastric Bypass vs. Duodenal Switch with Sleeve Gastrectomy in Rat Models
Source: PLoS One. 2013 Sep 9;8(9):e72896. doi: 10.1371/journal.pone.0072896 (PMC3767664; doi:10.1371/journal.pone.0072896)

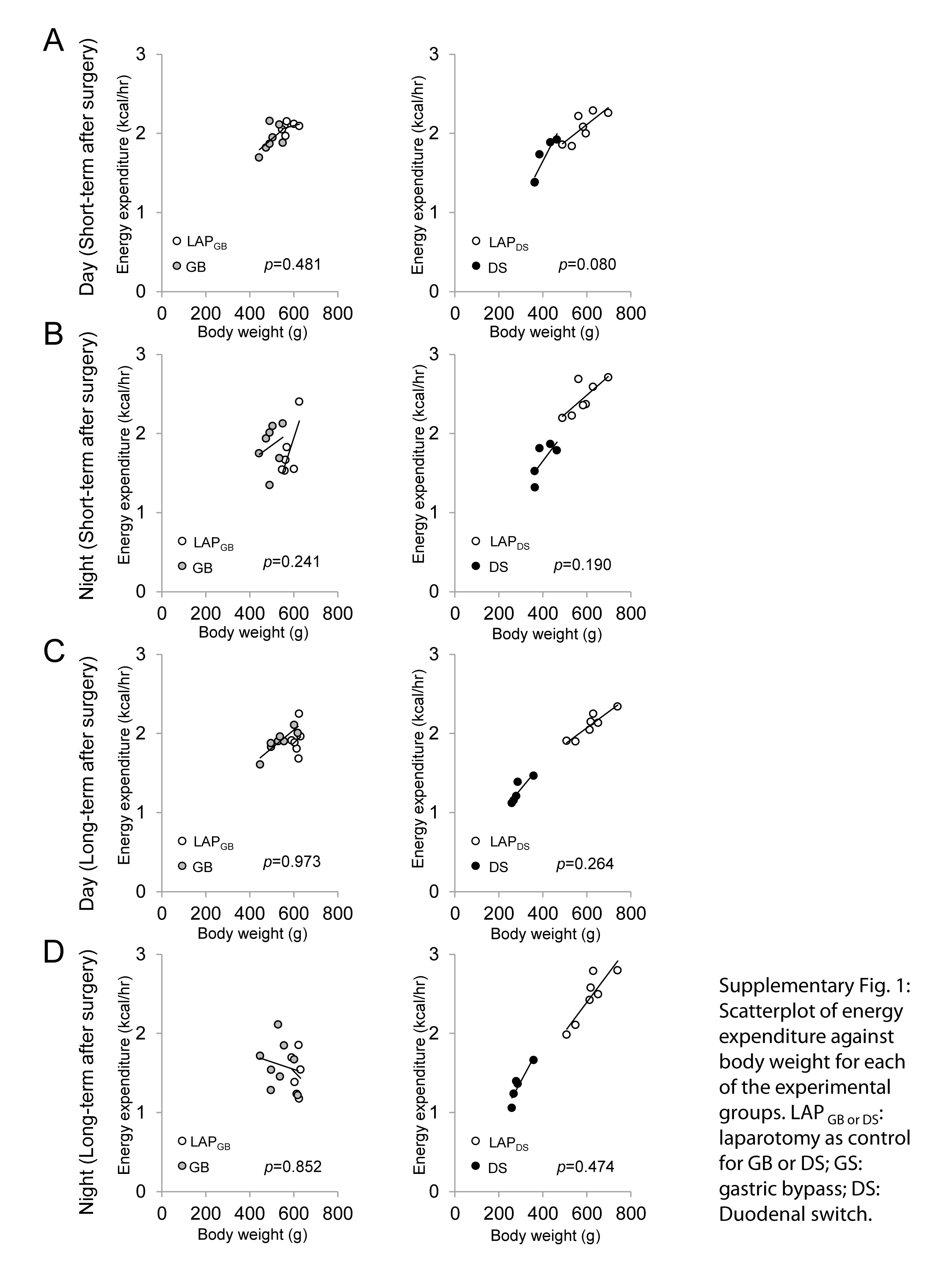

Supplement: Figure S1 — Scatterplot of energy expenditure against body weight. LAPGB or DS: laparotomy as control for GB or DS; GS: gastric bypass; DS: Duodenal switch. (TIF) [file pone.0072896.s001.tif]

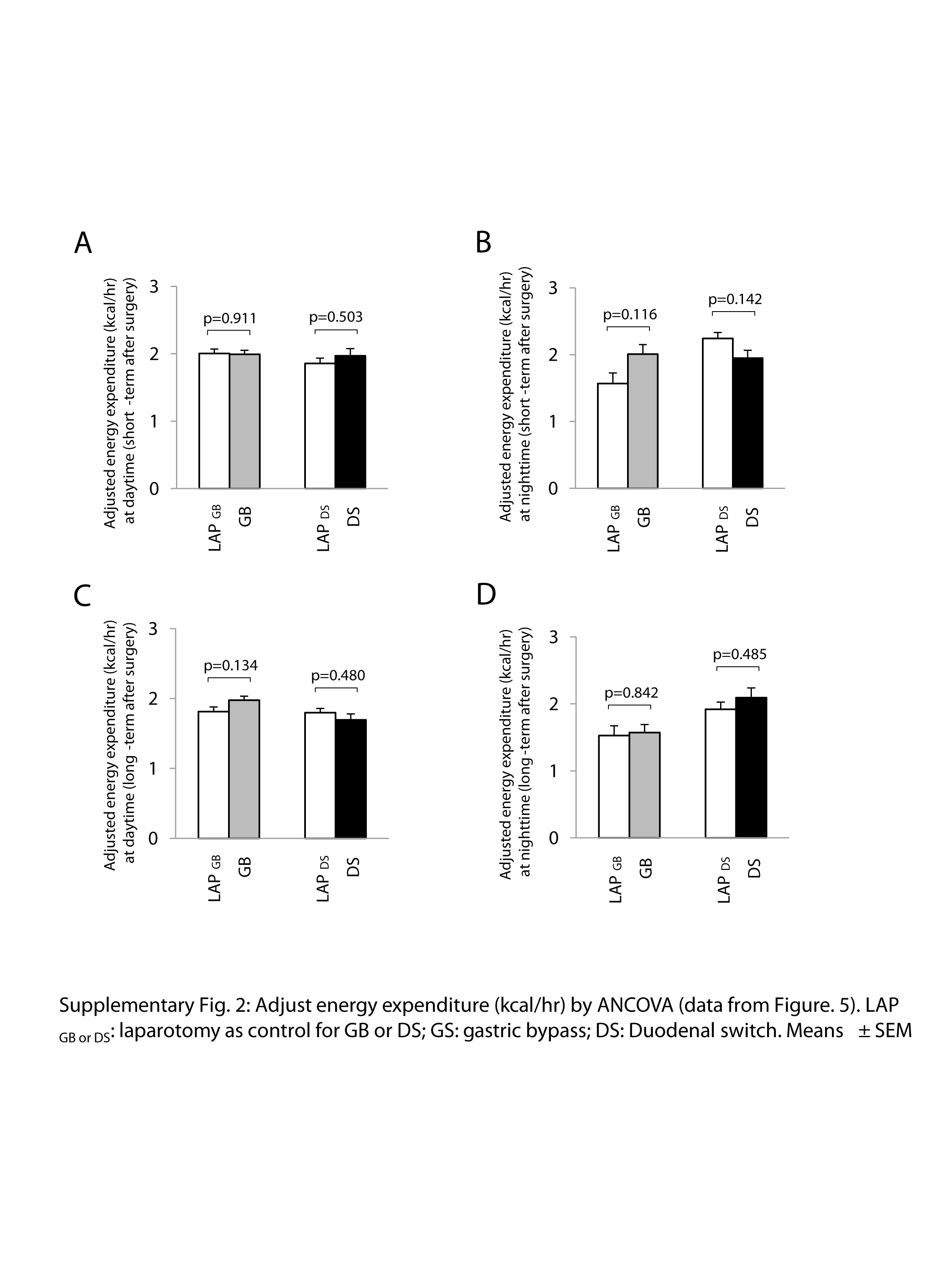

Supplement: Figure S2 — Adjust energy expenditure by ANCOVA. LAPGB or DS: laparotomy as control for GB or DS; GS: gastric bypass; DS: Duodenal switch. Means ± SEM. (TIF) [file pone.0072896.s002.tif]
