# Supplementary material for: Mechanistic Comparison between Gastric Bypass vs. Duodenal Switch with Sleeve Gastrectomy in Rat Models
Source: PLoS One. 2013 Sep 9;8(9):e72896. doi: 10.1371/journal.pone.0072896 (PMC3767664; doi:10.1371/journal.pone.0072896)
Supplement: Table S1 — CLAMS measurements of normal rats. Data at day 1 and 21 one week after 24 hours training with CLAMS cage are expressed as means ± SEM. ns: not significant between day 1 vs. day 21. (DOC) [file pone.0072896.s003.doc]

**Supplementary Table 1**. CLAMS measurements of normal rats at day 1 and 21 one week after 24 hours training with CLAMS cage. Data are expressed as means ± SEM. ns: not significant between day 1 *vs.* day 21.

|  |  |  |  |
| --- | --- | --- | --- |
|  | **Parameter** | **Day 1** | **Day 21** |
| **Day** | Food Intake (g) | 6.76±0.67 | 7.36±0.60 ^ns^ |
|  | Food Intake (g/100g body weight) | 1.38±0.14 | 1.46±0.14 ^ns^ |
|  | Food intake (kcal) | 17.36±1.72 | 18.90±1.53 ^ns^ |
|  | Food intake (kcal/100g body weight) | 3.54±0.35 | 3.74±0.36 ^ns^ |
|  | Number of meals | 14.17±1.66 | 13.50±1.48 ^ns^ |
|  | Meal size (g/meal) | 0.52±0.10 | 0.56±0.05 ^ns^ |
|  | Meal size (kcal/meal) | 1.34±0.25 | 1.45±0.12 ^ns^ |
|  | Meal duration (min) | 18.98±2.07 | 21.10±1.95 ^ns^ |
|  | Meal duration (min/meal) | 1.45±0.27 | 1.61±0.15 ^ns^ |
|  | Intermeal interval (min) | 49.44±5.95 | 50.43±4.48 ^ns^ |
|  | Satiety ratio (min/g) | 101.03±9.37 | 90.73±6.44 ^ns^ |
|  | Water intake (mL) | 2.17±0.64 | 2.24±0.42 ^ns^ |
|  | Water intake (mL/100g body weight) | 0.45±0.13 | 0.45±0.09 ^ns^ |
|  | Water intake during one interval (mL/time) | 0.25±0.07 | 0.28±0.05 ^ns^ |
|  | Ambulatory activity | 1305.17±318.30 | 1451.17±326.57 ^ns^ |
| **Night** | Food Intake (g) | 19.38±0.72 | 21.07±0.78 ^ns^ |
|  | Food Intake (g/100g body weight) | 3.94±0.13 | 4.15±0.19 ^ns^ |
|  | Food intake (kcal) | 49.80±1.85 | 54.16±2.00 ^ns^ |
|  | Food intake (kcal/100g body weight) | 10.13±0.33 | 10.67±0.50 ^ns^ |
|  | Number of meals | 29.17±2.65 | 28.83±3.89 ^ns^ |
|  | Meal size (g/meal) | 0.68±0.04 | 0.79±0.09 ^ns^ |
|  | Meal size (kcal/meal) | 1.75±0.11 | 2.02±0.24 ^ns^ |
|  | Meal duration (min) | 62.62±3.01 | 63.66±2.10 ^ns^ |
|  | Meal duration (min/meal) | 2.21±0.15 | 2.36±0.24 ^ns^ |
|  | Intermeal interval (min) | 22.57±1.80 | 23.88±2.95 ^ns^ |
|  | Satiety ratio (min/g) | 32.98±1.22 | 30.20±1.05 ^ns^ |
|  | Water intake (mL) | 16.14±0.74 | 17.70±0.60 ^ns^ |
|  | Water intake (mL/100g body weight) | 3.28±0.12 | 3.48±0.13 ^ns^ |
|  | Water intake during one interval (mL/time) | 0.87±0.15 | 0.89±0.09 ^ns^ |
|  | Ambulatory activity | 5292.00±1166.98 | 4484.00±833.95 ^ns^ |
|  |  |  |  |
