# Supplementary material for: Mechanistic Comparison between Gastric Bypass vs. Duodenal Switch with Sleeve Gastrectomy in Rat Models
Source: PLoS One. 2013 Sep 9;8(9):e72896. doi: 10.1371/journal.pone.0072896 (PMC3767664; doi:10.1371/journal.pone.0072896)
Supplement: Table S2 — Plasma levels of cytokines. Data of rats after gastric bypass (GB) and duodenal switch (DS) compared with the age-matched laparotomy-operated groups (LAPGB or LAPDS, respectively) are expressed as means ± SEM. ns: not significant between LAPGB vs. GB or LAPDS vs. DS. (DOC) [file pone.0072896.s004.doc]

**Supplementary Table 2.** Plasma levels of cytokines in rats after gastric bypass (GB) and duodenal switch (DS) compared with the age-matched laparotomy-operated groups (LAP_GB_ or LAP_DS_, respectively). Data are expressed as means ± SEM. ns: not significant between LAP_GB_ *vs.* GB or LAP_DS_ *vs.* DS.

|  |  |  |  |  |  |
| --- | --- | --- | --- | --- | --- |
| **Cytokine** | **LAP_GB_** | **GB** |  | **LAP_DS_** | **DS** |
| IL1α (pg/mL) | 66.20±21.31 | 33.48±21.01 ^ns^ |  | 94.34±33.31 | 124.84±19.20 ^ns^ |
| IL-1β (pg/mL) | 74.06±22.89 | 32.63±11.51 ^ns^ |  | 310.22±175.16 | 215.53±87.70 ^ns^ |
| IL-2 (pg/mL) | 201.62±59.23 | 112.19±46.99 ^ns^ |  | 190.21±40.10 | 373.21±69.13 ^ns^ |
| IL-4 (pg/mL) | 10.62±2.48 | 27.33±22.06 ^ns^ |  | 39.37±17.56 | 41.69±9.90 ^ns^ |
| IL-5 (pg/mL) | 38.08±8.80 | 41.19±10.48 ^ns^ |  | 129.45±26.03 | 106.05±18.71 ^ns^ |
| IL-6 (pg/mL) | 111.53±51.69 | 208.33±176.17 ^ns^ |  | 216.85±82.11 | 158.36±49.23 ^ns^ |
| IL-10 (pg/mL) | 213.33±29.71 | 145.88±31.72 ^ns^ |  | 534.60±139.86 | 588.50±74.00 ^ns^ |
| IL-13 (pg/mL) | 19.52±8.16 | 4.47±1.23 ^ns^ |  | 61.05±33.76 | 55.41±19.65 ^ns^ |
| GM-CSF (pg/mL) | 9.42±4.59 | 1.23±0.41 ^ns^ |  | 41.06±24.68 | 30.81±12.69 ^ns^ |
| IFNγ (pg/mL) | 53.90±25.31 | 106.47±105.90 ^ns^ |  | 76.90±38.39 | 75.45±24.11 ^ns^ |
| TNFα (pg/mL) | 9.25±6.98 | 3.72±0.35 ^ns^ |  | 65.66±46.06 | 56.94±29.70 ^ns^ |
|  |  |  |  |  |  |
